# University Day

**Published:** 1909-04

**Authors:** 


### University Day

THE faculty and students of the University of Buffalo, assisted by ministers, lawyers, doctors, dentists, city, county officials and others, held the annual University Day exercises at the Teck Theater February 22, 1909. The exercises form the university's celebration of Washington's Birthday, chosen University day ten years ago. Various patriotic organisations, as well as the students of the four high schools of Buffalo, joined in the celebration. The orator of the day was the Reverend Dr. Andrew V. V. Raymond, pastor of the First Presbyterian Church.

The students, including those from the high schools, marched from the university buildings at High street, down Main to Chippewa, then back to the Teck. The academic procession into the theater was led by Vice-Chancellor Charles P. Norton, who escorted the Reverend Dr. Raymond to the stage. The stage and other portions of the theater were decorated with flags and bunting and pictures of Washington.

The Reverend Dr. F. A. Kahler delivered the invocation, after which Mr. Norton introduced the Reverend Dr. Raymond. Dr. Raymond's address was on The Relation of Higher Education to the Life of the City.

The speaker characterised Washington as America's champion of justice and liberty. He said that it was proper upon the celebration of the anniversary of his birth to dwell upon the principles underlying American institutions; liberty and equality of opportunity.

"A larger life is the inalienable right of every man," said Dr. Raymond. "Life itself answers the question as to the natural end or aim of living,—namely, increase, more life, abundant life. The struggle should not be simply to live, but to unfold all the latent energies of life. I desire to emphasise this; that a man's business or profession is not the great end for which he is living, but the real end is the largest possible development of himself as a man.

"Education is the essential development of the ability to appreciate and choose the best things," said the speaker. "We are told that education, that religion must be practical nowadays and that only practical men can meet the demands of the present time. By the term practical, we generally understand that if a man lives so that he is able to build a fine house, to furnish it magnificently, if he lives so as to leave a fine fortune and provide in his will for a fine monument to be built on the finest lot in the finest cemetery in the city; if he lives such a life, he is a practical man.

"Then what a fool Lincoln was, if that be true, to live so that the only thing he could leave was a name and the only fortune was the love and gratitude of a nation. But what is greater than a name, I ask? What more practical man ever stood on this land of ours than Washington, leaving only a name, but a name that lives through generations."

---

THE first class of Filipino physicians trained under the American rule received their degrees from the Philippine Medical School at Manila, February 27, 1909. The dean of the faculty, Dr. P. C. Freer, formerly of the University of Michigan, presided over the commencement and addresses were delivered by commissioners D. C. Worcester and Newton W. Gilbert and Senor Rafael Palma, a member of the Philippine Assembly, Forty Filipino women who are studying to become trained nurses attended the ceremony.

---

THE committee appointed last year by the Medical Society of the State of New York to act jointly with a committee representing the State Bar Association to investigate the evils growing out of the employment of medical experts, under the present methods, reported back to the society at its recent annual meeting that the law should authorise the appellate divisions to appoint not less than ten or more than sixty physicians in the different judicial departments, who shall be qualified to act as medical experts, and that the expense of their service shall be borne by the county in which the action is tried.

---

IN 1908, the total number of bodies disposed of by cremation in Germany was *The British Medical Journal* says, 4,050, as against 2,977 in 1907, showing an increase of 1,073, or 36 per cent. Among those whose bodies were cremated were 1,474 women. The classification according to religious creeds gives some interesting results. While the majority of persons cremated were described as Lutherans, there was a considerable body of Catholics, notwithstanding the prohibition issued by Leo XIII. For some reason, in Germany, as in France, cremation does not seem to appeal to Free Thinkers. In 2,517 cases, all coming under the head of Lutherans, the incineration was accompanied by religious rites.

---

The Academy of Sciences at Vienna has decided upon the creation of phonographic archives, which will be divided into three parts, and which will probably be the most remarkable library on record. The first section will be devoted to examples of European languages and dialects of the different peoples spoken at the beginning of the twentieth century. The second will contain examples of music and song of the same period, while the third section will be reserved for the records of contemporary orators.

---

Professor Greef, director of the Berlin eye hospital, announced at Berlin, March 26, 1909, the discovery of the trachoma germ. He says his experiments with the germs on anthropoid apes convinced him that trachoma is contagious in its early stages.

---

An educational campaign by means of speeches and posters is being carried on under the direction of the Chicago Tuberculosis Institute in support of the question to be submitted to the voters at the next election to determine whether or not the citizens agree to a tax being levied for the establishment and maintenance of sanitoria for consumptives. Physicians who are desirous of establishing the institutions will go on the platform before various organisations and at meetings and will explain why the people should vote "Yes" on the question. Posters are being distributed in every section of the city in various languages pointing out the great loss of life annually in Chicago from the white plague.

---

The abolition of the Chappaqua Mountain Institute, in Westchester County, which was for years an excellent seminary of learning, will be regretted by many, but there will be consolation in knowing that its buildings and its particularly beautiful and salubrious site are to be used as a suburban sanatorium by the Children's Aid Society, under the generous endowment given by Mrs. Elizabeth Milbank Anderson. The establishment of such an institution will materially enlarge and facilitate the good work of that society, and it could probably not have been effected in a more desirable place than among the mountains and forests of Chappaqua.

Navy to have Medical Reserves.—The Secretary of the Navy has approved the recommendation of the surgeon-general for the establishment of a naval reserve medical corps similar to that recently organised in the army. This is to be in connection with the development and reorganisation of the naval militia, and was among the recommendations made by Surgeon-General Rixey this year. It is purposed to establish the reserve corps on the same basis as that branch of the army medical department, and to commission in the navy medical reserve corps the provisionally qualified candidates who are examined with a view to appointment in the regular medical corps of the navy. By this means they will serve a probationary period, during which they will be specially instructed at the Naval Medical School, in Washington. This branch will enable the Navy Department to obtain contract surgeons to assist the regular doctors at naval hospitals, and will afford the means of expanding the medical corps in time of war.

